# Myocardial conduction network visualized by magnetic resonance microscopy/diffusion imaging and validated by histology

**DOI:** 10.1186/1532-429X-17-S1-O73

**Published:** 2015-02-03

**Authors:** John R Forder, Min-Sig Hwang, Stephen J Blackband

**Affiliations:** 1 Radiology, University of Florida, Gainesville, FL, USA; 2 University of Pittsburgh, Pittsburgh, PA, USA; 3 Neuroscience, University of Florida, Gainesville, FL, USA

## Background

Magnetic resonance microscopy (MRM), combined with high resolution diffusion imaging, has been used to identify tractography that corresponds to the known distribution of cardiac conducting fibers in the left ventricle. The conducting network transits through the myocardial tissue in different orientations, and with different characteristics. In order to validate that the observed fibers calculated from the diffusion data correspond to cardiac conducting fibers, we performed immunohistochemical staining on distinct regions of the myocardium.

## Methods

Hearts were isolated from New Zealand White male rabbits, and retrograde perfused with a Krebs Henseleit solution to maintain viability and remove red blood cells. The perfusate was switched to a St. Thomas Hospital cardioplegic solution to arrest the heart, and then perfusion fixed with 3% formalin solution. Hearts were immersion fixed as well for 72 hours. A section of the left ventricular wall corresponding to the location of the cardiac conducting bundle (approximately 5mm x 7mm x 24mm) was dissected free and prepared for imaging and histology.

A 750MHz (17.6T) wide bore spectrometer (Bruker Instruments, Billerica, MA) was used to acquire MRM images at 35µm^2^ in-plane resolution with a 82µm slice thickness for anatomical information, and high angular resolution diffusion imaging (HARDI) was acquired using 50µm^2^ in-plane resolution and a 400µm slice thickness. Diffusion gradients were oriented in 21-directions, using a nominal b-value of 1000.

Following the imaging, the same sample underwent histological staining. Samples were stained with H&E, and with immunohistochemical staining for neurofilament. Images from the histological imaging were registered with the MRM and HARDI data sets for comparison.

## Results

**Figure 1:**
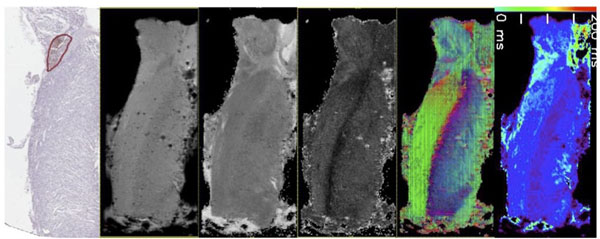
Ventricular septum sectioned long axis. Panels (from left to right): 1) Immunohistochemical staining for neurofilament (circled area corresponds to cardiac conducting bundle), 2) S_0_ image (absence of diffusion gradients), 3) apparent diffusion coefficient image, 4) generalized anisotropy, 5) principal eigenvector, and 6) T_2_ map. The T2 map delineates the same region identified in the neurofilament staining.

**Figure 2:**
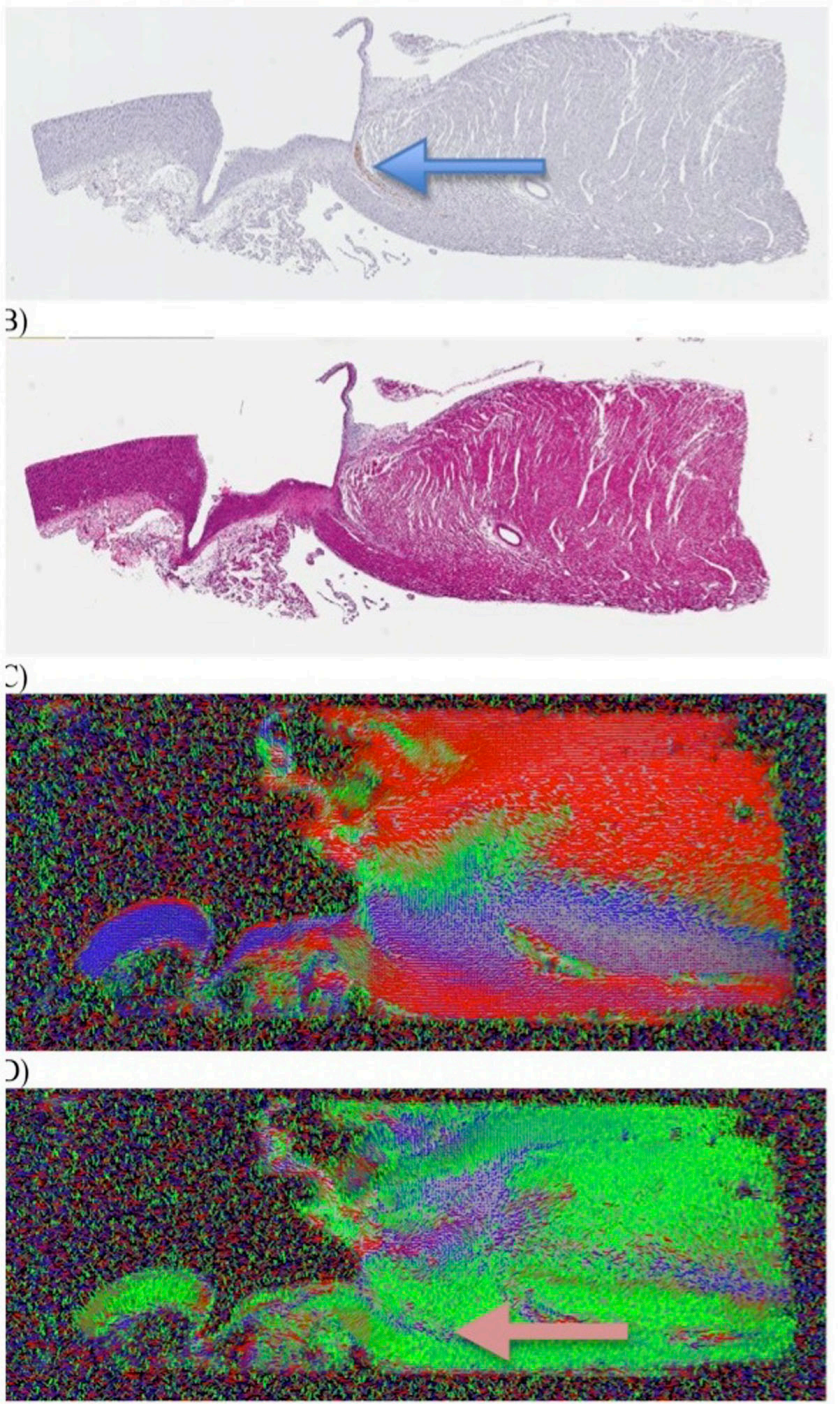
Histology and HARDM data shows 2° eigenvector reflecting cardiac conducting bundle. Panel A shows section of LV septum (oriented l-r => base to apex) stained for neurofilament. Arrow points to conducting bundle. Panel B is the same tissue with H&E stain, with no defining characteristics. Panel C is a map of the primary eigenvector for the same region, while panel D is the secondary eigenvector. Although the primary eigenvector for the conducting network is hidden in adjacent pixels with similar orientations, the secondary eigenvector appears to be specific for the conducting bundle.

Under conditions where the orientation of the conducting fibers was orthogonal (or nearly orthogonal) to the predominant orientation of the cardiomyocytes, the primary eigenvector of diffusion corresponded to areas that stained for neurofilament. When the conducting bundle is oriented along the same axis as the predominant cardiomyocyte direction, specificity is lost if using only the primary eigenvector (Figure 1). However, the orientation of the secondary eigenvector does correspond with the staining for neurofilament under these conditions (Figure 2).

Average diffusivity was not different between tissue types, but T2 maps showed good agreement with tissue staining.

## Conclusions

HARDI imaging at microscopic resolutions can image cardiac conducting fibers, but orientation of the fibers along the same directions as the muscle fibers presents challenges. Including T2 mapping and the secondary eigenvectors may provide improved sensitivity under these conditions.

## Funding

The authors would like to acknowledge the National Institutes of Health, National Heart Lung and Blood Institute, grant R56 HL122064-01 (Forder) for providing funds for this study.

